# Application of DNA parentage testing and EnVigour HX™ to evaluate bull prolificacy and heifer performance in beef cattle breeding programs in Western Canada

**DOI:** 10.1093/tas/txaf129

**Published:** 2025-09-23

**Authors:** Tess Mills, Razie Khorshidi, Daalkhaijav Damiran, Mika Asai-Coakwell, Diego Moya, Kathy Larson, Herbert A Lardner

**Affiliations:** Department of Animal and Poultry Science, College of Agriculture and Bioresources, University of Saskatchewan, 51 Campus Drive Saskatoon, SK S7N 5A8, Canada; Department of Animal and Poultry Science, College of Agriculture and Bioresources, University of Saskatchewan, 51 Campus Drive Saskatoon, SK S7N 5A8, Canada; Department of Animal and Poultry Science, College of Agriculture and Bioresources, University of Saskatchewan, 51 Campus Drive Saskatoon, SK S7N 5A8, Canada; Department of Animal and Poultry Science, College of Agriculture and Bioresources, University of Saskatchewan, 51 Campus Drive Saskatoon, SK S7N 5A8, Canada; Department of Large Animal Clinical Sciences, Western College of Veterinary Medicine, University of Saskatchewan, 51 Campus Drive Saskatoon, SK S7N 5B4, Canada; Department of Agricultural and Resource Economics, College of Agriculture and Bioresources, University of Saskatchewan, 51 Campus Drive, Saskatoon, SK S7N 5A8, Canada; Department of Animal and Poultry Science, College of Agriculture and Bioresources, University of Saskatchewan, 51 Campus Drive Saskatoon, SK S7N 5A8, Canada

**Keywords:** beef cattle, crossbreeding, parentage testing, genomic breed composition, heifer performance, sire prolificacy

## Abstract

The use of multi-sire breeding pastures is a common practice in Western Canada for beef cattle management. However, the number of progenies sired by each bull may not be known or producers will not be sure if performance traits were passed on. Hybrid vigor is defined as the superiority of crossbred progeny over their parents’ average to increase production efficiency, longevity and reproductive rate of beef cows. A DNA parentage test was performed to identify relative sires to their progeny. Then, 109 bull prolificacy indexes (BPI) were calculated for 46 sires over 6 breeding seasons (some had more than one BPI). They ranged from 0.04 to 3.47, with values larger than one showing high prolific sires. Yearling sires had a significantly lower BPI value than 2-year-old and mature bulls. Regardless of the level of BPI, each sire produced the majority of all his born calves in the first cycle of the calving period (R^2^ = 0.89). This demonstrated the importance of identifying high BPI sires, as they showed a tendency to have a greater percentage of the first cycle born grand calves from their first cycle born heifers (R^2^ = 0.86). Also, they produced a greater number of grand calves from their retained daughters with a significant impact on total kg weaned (P < 0.01). The EnVigour HX™ test was applied to estimate the effect of bulls’ and heifers’ vigor scores (scores were based on percentage) on fertility and production traits. The breeder’s interest in the utilization of crossbred heifers and bulls positively affected the longevity of replacement females. There was a decrease in age at first calving relative to the EnVigour HX™ test commercialized suggestions per 10% increase in heifers’ vigor scores. Moreover, larger productivity was observed for average WW per calf and cow life productivity, which could be due to different genomic breed compositions between this study and the test animals. Overall, DNA parentage and EnVigour HX™ tests are essential tools for beef cattle production profitability. However, up to a 75% vigor score cutoff is suggested when selecting crossbred females with EnVigour HX™ to retain production and fertility efficiency in crossing with Beef Booster bulls.

## Teaser

DNA parentage test is essential to determine prolific bulls in multi-sire breeding pastures.

Increasing hybrid vigor score of crossbred females above 75% will not meaningfully extend production and fertility efficiency when crossed with Beef Booster bulls.

## Introduction

Cattle breeding programs are one of the most important considerations for a ranch, as the economic potential of a ranch is highly related to the herd’s reproductive performance ­(Chenoweth, 2011). While proper health and management of breeding animals are important for a producer’s economic benefit, it is also important to improve herd efficiency overall to be able to feed a growing population (Thundathil et al., 2016). In Western Canada, the use of multi-sire breeding pastures for beef cattle is a common practice, especially with industry consolidation leading to increasing herd sizes (Makarechian et al., 1987; Tang et al., 2011). However, in this system, producers may not know for certain which progeny are sired by each bull, and they will not be able to determine if performance traits were passed on (Van Eenennaam et al., 2007).

According to the Canadian Cow-Calf Cost of Production Network, the sale of weaned calves is the primary source of income for cow-calf producers, which ranged from 70-85% of enterprise revenues in 2022 (Canfax, 2020). Herd sires need to be effective breeders to sire as many offspring as they can for cow-calf producers to generate revenue. There is an increasing interest in tracing sires for marketing feeder cattle with enhanced genetic merit in branded programs. Seedstock producers use genomic testing and Expected Progeny Difference (EPDs) to identify sires with superior traits, allowing to market them to commercial cow-calf operations seeking consistent quality and higher profits. Most cow-calf producers retain heifers to develop into replacement breeding females (USask, 2018; USDA 2008). Therefore, the more calves a bull sires, the more he contributes to the genetic direction of the breeding females on the ranch (Van Eenennaam et al., 2014).

When multiple sires are used in breeding fields, the expectation is that each bull will sire an equal proportion of calves. Previous research has shown this expectation is typically not met; a disproportionate number of calves are often sired from one bull, while another bull may only sire one calf (Abell et al., 2017; Domolewski, 2023). This can be influenced by a variety of behavioral effects on the bull, including the bull’s libido (Blockey, 1979). Libido tests are no longer conducted, leaving producers to rely on breeding soundness evaluations to determine if a bull is fit for breeding. With DNA parentage testing technology, a producer can verify a bull’s contribution to the calf crop and eliminate low producing bulls not economically beneficial to the operation (Holroyd et al., 2002).

Heterosis or hybrid vigor is defined as the superiority of performance of crossbred progeny over their parents’ average. In commercial beef production sector, using crossbred cattle over purebred ones for the exploitation of heterosis and breed complementarity has the benefits of increasing overall production efficiency, longevity and reproductive rate of beef cows (Cundiff et al., 1992; Núñez-Dominguez et al., 1992; MacNeil et al., 1994). This will become important when producers select which bulls to mate with which females because it can impact the herd improvement for multiple generations, especially when replacement females are selected from within the herd (Wiggans et al., 2017; Ortega et al., 2018). Today, single nucleotide polymorphism (SNP) panels provide innovative genomic tools to predict genomic breed composition of crossbred animals and their genomic vigor score (genomic retained heterozygosity which is positively linked to heterosis) to optimize parents’ mating plans and help produce future progeny with improved performance (Akanno et al., 2017). As an example, EnVigour HX™ is a genomic test developed by Delta Genomics^®^ (Edmonton, Alberta, Canada) to help beef producers understand the animal’s hybrid vigor and develop an optimal crossbreeding strategy for their herd (Delta Genomics, 2018).^1^

Few studies have examined the variation of calves sired in multi-sire breeding pastures and evaluated the consistency of an individual bull’s performance over time. Also, commercially available genomic tools are in short supply for selecting bulls with superior breeding performance that would be passed along to male or female progeny. Therefore, the objectives of this study were 1) to evaluate the advantage of DNA paternity testing in multi-sire breeding groups to measure the repeatability of sire’s contribution through progeny number per year, and assess the performance of sires’ daughters over multiple parities and 2) to examine the effectiveness of EnVigour HX™ genomic test in evaluating the prolificacy of crossbred bulls and the performance of their crossbred heifers on fertility and production traits.

## Materials and Methods

### Study site and animal management

Data collection involved collaboration with a commercial cow-calf ranch operation located in the province of Saskatchewan over a 6-year period and a genomic testing company in Edmonton, Alberta. The ranch operation was in southwest Saskatchewan in the Mixed Grassland ecoregion of Prairies Ecozone (AAFC, 2023) and Brown soil zone. Data was collected in cooperation with the herd owners for the 2014-2021 breeding seasons. The cows started calving in April each year. A total of 46 bulls were used over 6 breeding seasons (2014-2019), with 20 bulls being used for 3 or more years. The herd females were crossed with Red Angus, Black Angus, and mostly Beef Booster crossbred bulls. This was because the Beef Booster breeding scheme was based on three maternal strains (M1, M2 and M4) and two specialized sire lines (M3 and TX) (MacNeil et al., 1994). The base for M1 was Angus breed to produce and raise a superior calf and M2 was Hereford to be well adapted to North America climates, along with including dairy breeds such as Holstein for M1 and Brown Swiss for M2 to improve the milking ability and udder of these strains. For more growth and milk production, Limousin and Simmental were also used in M1 and M2 (De Boer, 1984). The suitable bases for the M4 strain with the all-purpose cattle objective were Gelbvieh and Limousin. M3 was an Angus-Hereford base to achieve light birth weight on heifers to minimize dystocia and calf mortality with the introduction of Jersey and Shorthorn to give a breed of good milk production with easy-calving capacity (De Boer, 1984). The TX was a terminal sire strain designed for fast-growing feeders to yield a desirable carcass after slaughter including Maine Anjou, Charolais and Chianina (De Boer, 1984; MacNeil et al., 1994).

Each year there were 4 multi-sire breeding groups: 2 groups of mature cows (group 1 and 2, > 3 years of age), 1 group of 3-year-old cows (group 3), and 1 group of replacement heifers (group 4, 2-year-olds) (Table 1). The ages of bulls and cows were recorded each year from producer records. Bulls and cows were not assigned to the same breeding group each year but were assigned based on the judgment of the producer. Bulls were removed from the breeding pasture if injured and typically replaced with a spare from the replacement group. Each year, groups 1 and 2 had the highest number of bulls (5-8) and groups 3 and 4 had the least number of bulls (2-4) (Table 1). Breeding season was between July and September, with the calving season spanning 69-85 days. All management and procedures involving live animals followed the guidelines outlined in the Canadian Council on Animal Care (CCAC, 2009).

**Table 1.** Descriptive statistics for each breeding group by year, bull to cow ratio, sire age mean and corresponding BPI range.

Breeding group (type)	Breeding year	Sire No.	Cow No.	Bull:Cow ratio	Mean sire age (year)	Total No. of calves^1^	Min No of calves	Max No of calves	Min BPI	Max BPI
**1**	2014	5	130	1:26	2	126	10	43	0.4	1.7
**2**		6	136	1:23	3.9	121	4	51	0.2	2.5
**3**		3	67	1:22	2.5	61	14	28	0.7	1.4
**4**		4	75	1:19	2.5	66	12	21	0.7	1.3
**1**	2015	6	162	1:27	2	134	8	51	0.4	2.3
**2**		6	154	1:26	2.7	136	1	38	0.04	1.7
**3**		3	70	1:23	3.7	57	4	33	0.2	1.7
**4**		4	85	1:21	2.1	37	2	19	0.2	2
**1**	2016	7	172	1:26	2.4	141	4	52	0.2	2.6
**2**		6	159	1:27	2.2	115	1	49	0.04	2.1
**3**		2	39	1:20	4.8	32	5	27	0.3	1.7
**4**		3	71	1:24	2.5	58	27	31	0.9	1
**1**	2017	8	154	1:19	2.3	95	4	36	0.2	1.9
**2**		6	159	1:27	2	82	12	33	0.6	1.6
**3**		3	71	1:24	3.4	49	8	27	0.5	1.6
**4**		3	78	1:26	2.9	49	1	29	0.06	1.8
**1**	2018	8	184	1:23	2.9	113	1	49	0.07	3.5
**2**		7	148	1:21	3	104	2	24	0.1	1.6
**3**		3	79	1:26	2	54	4	26	0.2	1.4
**4**		3	79	1:26	4.3	63	2	50	0.1	2.4
**1**	2019	7	157	1:22	2.8	115	2	66	0.1	3.4
**2**		7	157	1:22	3	111	5	42	0.3	2.3
**3**		4	79	1:26	2.3	68	1	27	0.06	1.6
**4**		4	90	1:23	3	80	4	42	0.2	2.1

1 Only includes calves with a matched sire. Any calves with no sire match were not included in the total for this column.

From 2014 to 2018 the herd was owned and managed by one owner, then sold to a new owner/management in 2019. Replacement heifer selection criteria changed slightly from 2018 to 2019 due to ownership and management changes. All management decisions were made by the owner and the research study did not have an influence on any management decision making. Between 2015 and 2018 a heifer was ineligible for selection as a replacement if: a) she was a twin with a bull; b) she had been treated with antibiotics as a suckling calf; c) her dam’s weight exceeded 771 kg; d) her behavior in chute was a concern; or e) she was a daughter of an unwanted bull. After culling, heifers were weighed, and the top 90 females were retained. When the herd changed ownership in 2019, selection criteria included culling heifers: a) from temperamental dams; b) with poor conformation (including if a heifer’s dam had a poor udder, feet or body); c) born in the third cycle; or d) low body weight at weaning.

### Data and sample collection

Each year at calving, notches of ear tissue were collected from all calves, both live and dead, using a specialized Tissue Sampling Unit (TSU) tagger by Allflex^®^ (Allflex, 2021). The samples were either frozen or stored at room temperature depending on the vial used for collection, which varied by year. They were then couriered to a lab for DNA parentage testing. Each ear tissue sample was related to a calf ear tag identification number and a list of potential sires based on the dam’s breeding field. From all bulls used for breeding, ∼20 tail hairs with follicles attached were collected once per animal for lab analysis at a time when the bulls were brought to the chute for processing (McGrath, 2014). All samples from 2015 to 2017 were sent to Quantum Genetix in Saskatoon, Saskatchewan, while samples from 2018 to 2020 were sent to Delta Genomics (now Neogen Canada) in Edmonton, Alberta for DNA parentage testing. All DNA samples were analyzed using SNP technology for sire verification. At Quantum Genetix, analysis followed the procedure outlined by Domolewski (2023) for all samples prior to 2018. qRT_PCR genotyping reactions were run as a multiplex to reveal the status of four alleles per reaction (two of one SNP and two of another SNP). Since information from 100 SNPs is used in sire verification, a minimum of 50 multiplex reactions were run per DNA sample. Additional SNPs were tested when a correct sire could not be identified using the 100 SNP panel, and if a sire still could not be determined, the calf was classified as ‘no sire match’ (Domolewski, 2023). Similar testing was performed for all samples submitted from 2018 onward at Delta Genomics lab. Calf birth date and dam and calf identification were recorded at calving each year based on dangle tag number and letter year (total number of calves tested = 2374, total number of calves with sires verified = 2234). Calf birth weights were obtained within 24 hours of birth and weaning weights were obtained in October each year, at approximately 172 days of age.

Heifers and calves born in 2018, along with 4 Angus and 16 Beef Booster bulls were analyzed for genomic breed composition and vigor score by the EnVigour HX™ test. Their DNA samples were genotyped on Illumina Bovine 50K SNP panels. These sires were used in crosses with all the dams and the 2018 born heifers.

### Calculations and statistical analysis

An index called the Bull Prolificacy Index (BPI) was calculated for each sire in every specific breeding year using the equation reported by Domolewski (2023) to determine BPI variation across years:


$$BPI=\frac{n}{\left(\frac{N}{m}\right)}$$

where BPI for each breeding year is calculated with the number of calves (n) attributed to a sire through genomic parentage testing divided by the ratio of the total number of calves born (N) to the total number (m) of sires in the breeding pasture. To correct for BPI based on calves born in the first 21 days of calving, n and N were substituted with the relative number of calves born in this cycle. Based on the guidelines established for Standardized Performance Analysis (SPA) in the University of Nebraska-Lincoln, the start date of the first 21-day calving was determined when the 3^rd^ mature cow (3 yr-old and older) calves. The BPI index allows for standardized comparison across breeding groups per year by accounting for different bull to cow ratios and pregnancy rates. An index value of 1 means a bull sired the “expected” number of calves (e.g., an equal proportion) based on total bulls in the breeding group and number of calves born and parentage verified. A score above 1 means the bull exceeded expectations and sired a higher number of calves than expected. A score below 1 means the bull sired fewer calves than expected for that breeding season. It is more accurate to use BPI, when analyzing sire performance, over total calves sired because calves attributed to a sire alone does not account for cow pregnancy rate or bull to cow ratio. BPI represents the proportion of a sire’s contribution based on their expected contribution and the opportunity to contribute.

Statistical analysis was performed using SAS 9.3 (SAS version 9.3; SAS Institute Inc., Cary, NC) to evaluate sire age influence on BPI analysis. The ANOVA procedure of SAS was analyzed using PROC MIXED to consider sire as a random effect (2020). The model was defined as follows:


$$Y_{ij}=\mu +{\mathrm{year}}_{i}+{\mathrm{age}}_{j}+(\mathrm{year}\times \mathrm{age})+e_{ij}$$

where Y_*ij*_ = response variable (continuous sire’s BPI); µ = mean; breeding year (year) and sire’s age (age) were both fixed effects; and error was *e_ij_*. Differences in means were determined to be significant when *P *< 0.05.

Each bull’s BPI score in a single year was used to sort the herd sires into three groups of Bottom 25%, Average 50%, and Top 25% BPI scores for further comparison of heifers’ performance. The coefficient of determination (R^2^) and/or Spearman correlation was calculated to determine if there is a relationship between bulls’ prolificacies and/or their daughters’ performance in consecutive breeding years.

Individual animal genomic-based breed composition was estimated through ADMIXTURE software (Alexander et al., 2009) to identify the proportion of each founder breed contribution and calculate the vigor score (or retained heterozygosity) of animals (Basarab et al., 2018). The EnVigour HX™ test can detect 13 different breeds, including Angus, Brown Swiss, Charolais, Galloway, Gelbvieh, Hereford, Holstein, Jersey, Limousin, Maine Anjou, Salers, Shorthorn, and Simmental. If an animal had evidence of a breed in addition to these 13 or portions that could not be determined to be of the above breeds, it was assigned a percentage “indeterminate”. The vigor score of each animal in the EnVigour HX™ tool is reported as a percentage from 0 to 100 or as a fraction of 0.0 to 1.0, where the lower the number, the less hybrid vigor the animal is deemed to have. Following Dickerson (1973), vigor score is calculated for each individual according to the following formula:


$$\mathrm{Vigor}\;\mathrm{Score}=1-\sum_{i=1}^{n}P_{i}^{2}$$

where P_i_ is equal to the fraction of each of the n contributing breeds in crossbred animals (Basarab et al., 2018). For example, for a crossbred animal with 50% breed A (P_1_ = 0.5), 25% breed B (P_2_ = 0.25) and 25% breed C (P_3_ = 0.25), the vigor score will be equal to 62.5%. If A is considered as the predominant breed, a gradual increase in its contribution from 50% to larger values (60, 70, and so on) that accordingly results in some reduction in breeds B and C proportions (the sum of three constructing breeds’ fractions should always be one) will lead to lower vigor scores and more tendency of animals to perform as predominant purebreds. Conversely, adding more breeds (4 to 8) to the construction of crossbred animals will reduce the contribution of the predominant breed to smaller than 50% and result in higher vigor scores and a tendency of animals to perform as crossbreds (Gregory et al., 1982).

For all dams born in 2015 and 2016 that were not genomically tested, the relative genomic breed compositions were averagely estimated using those 20 Angus and Beef Booster sires’ and their 2018 born progeny’s genomic breed compositions, ie, dam’s genomic breed composition = (2 × calf’s genomic breed composition—sire’s genomic breed composition). No genomic information was found for 2017 born heifers. Linear regression analyses were performed to compare the effects of increased vigor score on fertility and production traits with EnVigour HX™ test commercialized declarations (Delta Genomics, 2018). According to this test (Basarab et al., 2018), with every 10% increase in vigor score, there should be a 1.33 kg increase in wean weight (*P *= 0.23), females will be 2 days younger in age at first calving (AFC) (*P *= 0.03), they will have 2% higher conception rates (*P *= 0.04) and 35.7 kg increase in cow lifetime productivity (*P *= 0.02).

## Results and Discussion

### Bull prolificacy

Descriptive statistics based on breeding group per year of calf output are shown in Table 1. Most breeding groups had sires differing in age. There was variation in calf numbers between both breeding groups and individual sires within breeding groups. Calf number per sire differed each year and per breeding group and ranged from 1 to 66 across all six years. The maximum number of calves sired was influenced by bull performance, which was impacted by physical health, nutrition, bull to cow ratio, environment, and libido (Blockey, 1979; López et al., 1999). Sire prolificacy varied by group and individual bull across years (Table 1). Each year there was a large range in the BPI values of sires with at least one sire per group that achieved a BPI score above 1.0. Over 6 years, 109 BPI values were calculated for 46 sires, with a median of 0.93 and an average value of 1.00. The highest BPI value was 3.47 and the lowest was 0.04. Sires with a BPI at the bottom 25% had a BPI of less than 0.4, while bulls in the top 25% had a BPI greater than 1.4. Based on the BPI values from the current study, 0.4, or the bottom 25% of sires’ BPI, was arbitrarily determined to be the minimum value needed for sires that would be recommended to remain in the breeding group. The results of this study were consistent with previous research evaluating sire prolificacy (Van Eenennaam et al., 2007; Domolewski, 2023).

### Sire age

**Fig. 1.**
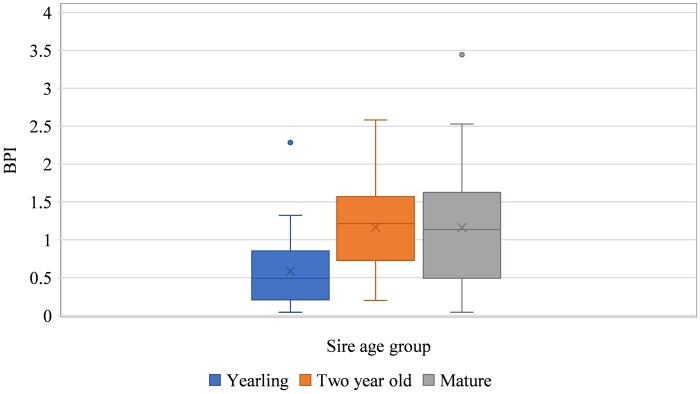
Range of BPI values based on sire age group over 6 years of breeding seasons.

The effect of year was found not significant for sire’s BPI in multiple years of use for breeding from 2014 to 2019. However, the age of sires had an influence on their BPI values. Sire age ranged from 1 to 6 years of age. Yearlings had significantly lower BPI (*P *< 0.05) values compared to 2-year-old and mature bulls (≥3 years, Figure. 1) with an average of 0.59 (SD = 0.50). Although there was a slight change in the range of BPI values between the 2-year-old (SD = 0.63) and mature bulls (SD = 0.81), no significant difference was observed between these two groups. They both showed similar median and mean BPI values of around 1.17, representing a sire that produced the expected number of calves after accounting for cow to bull ratio, conception and calving rate in the breeding group (Figure. 1). In a study by Van Eenennaam et al. (2014), they found no significant relationship between calf number and bull age, though bulls with higher age tended to sire a higher number of calves. Moreover, the results of this study were in line with Bennett et al. (2021) who observed a significant increase in prolificacy of bulls older than 2 years of age compared to younger animals.

Sires generally tended to have increased BPI values as a 2-year-old compared to yearling sires (Figure. 1). Moreover, most sires that exceeded the expected BPI (BPI > 1) as a yearling remained above the expected BPI as a two-year-old. There was a positive Spearman correlation of 60% (*P *< 0.01) for an increase in the level of BPI, suggesting bulls might tend to improve their BPI from year one to year two of breeding. Considering only genotyped Beef Booster bulls used in this herd, a higher meaningful Spearman correlation of 68% (*P *< 0.01) was observed between BPI in their first year of use with BPI in the second year of use. The higher fertility might be due to the higher vigor score measured in the Beef Booster bulls which will be discussed later. In addition, Spearman correlations of 50% (*P *= 0.04) and 57% (*P *= 0.11) were observed between sires’ BPI in their first year with the third and fourth years of use in breeding, respectively. If a sire’s performance is repeatable, it would be beneficial information for a producer to select for the top producing sires and cull the bottom producing ones. The conditions of the current study were like Van Eenennaam et al. (2014) sire repeatability calculations with a range higher than their reported values (0.33 to 0.37) for different ages. Holroyd et al. (2002) also found a similar range under their extensive Australian conditions at 0.43 to 0.69.

Age can influence sire performance in multiple ways. Reproductive development potential of sires can be influenced by early nutrition, age at puberty, sperm production potential, and testis size at maturity (Thundathil et al., 2016). Moreover, in the young and mature sire groups, the presence of a dominant or mature sire has been found to influence the prolificacy performance of other bulls by reducing their attempts at females nearby (López et al., 1999). Accordingly, regardless of age, if there is a strong, mature sire in a multi-sire breeding group, that could negatively impact the performance of the rest of the sires in that group.

### Sire performance based on calves born in the first 21 days of breeding cycle

**Fig. 2.**
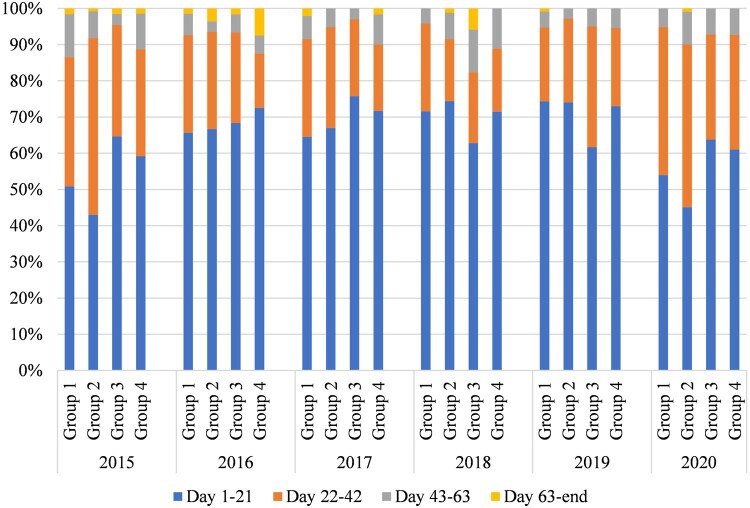
Calving distribution by percentage of calves born in each breeding cycle by breeding group per year.

Reproductive traits such as fertility, calving ease, libido, adaptability and structural soundness are important to consider, as they can drastically improve or reduce the number of calves produced in the first 21 days (Gutiérrez et al., 2002). Looking at all groups of cows and heifers together, from 2015 to 2019, there was a significant increase of 3% (*P *< 0.01) per year in the number of calves born in the first 21 days of calving with an overall average of 67% (Figure. 2). This might be because of the selection of higher vigor score bulls and females within each breeding group over time (though it was not directly mentioned by the breeder). In addition, there was some decrease observed in the number of 2018-2019 born calves of the first 21 days in the breeding group 3 and 2020 born calves in the groups 1 and 2, respectively. Though not meaningful, it might be due to the integration of pure Angus bulls and lower vigor score females in the mating plans of these groups. It is recommended that 60% of the calf crop is born in the first 21 days based on improvements in fertility and longevity for the cows and positive influences on uniformity and increased weight gain for calves by weaning (Cushman et al., 2013; Damiran et al., 2018). In this study involving multiple breeding groups, the 60% of calves born in the first 21 days benchmark was almost achieved every year, except for groups 1 and 2 in the years 2015 and 2020, respectively. Similar to the year 2020, the year 2015 also showed the inclusion of Angus bulls and lower vigor score females. Further discussion of the vigor score of animals will be provided later. Producing a calf in the first 21 days is especially important for heifers to ensure they have enough time to recover and cycle again to maintain a 365-day calving cycle and increase longevity in the herd (Damiran et al., 2018).

**Fig. 3.**
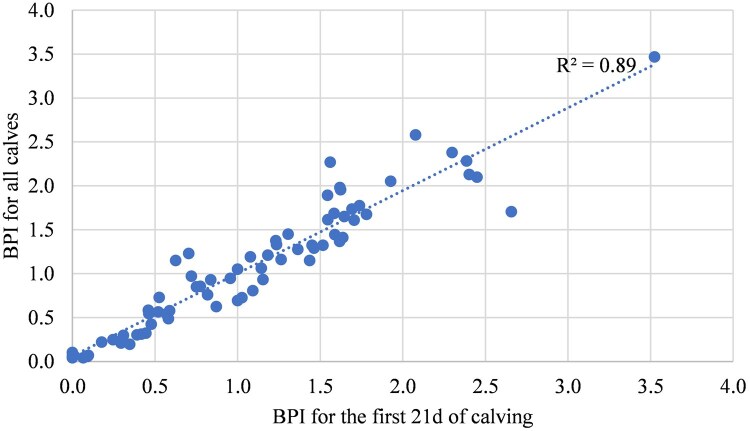
The relationship between sires’ BPI for all calves versus the first 21d born calves for bulls used for ≥ 3 years.

Looking at Figure. 3, comparing bulls with ≥ 3 years of BPI scores revealed that there was a direct relationship (R^2^ = 0.89) between their BPI for all and the first 21 days born calves. In fact, regardless of the level of BPI as low to high scores for multiple years, each sire produced the most of his calves in the first cycle of calving period. These results might suggest how prolific sires can impact the frequency of calves born in the first breeding cycle.

**Fig. 4.**
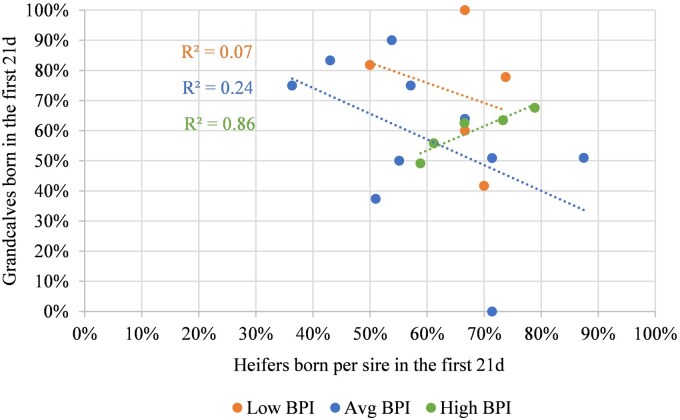
The relationship between heifers born in the first breeding cycle and their subsequent first 21 days born progeny on a per sire BPI basis.

Figure. 4 depicts the relationship between the percentage of 2015 to 2017 born replacement heifers sired in the first cycle and the percentage of grand calves also born in the first 21 days from 2017 to 2020, for 20 sires representing three BPI categories (25% Low, 50% Average, 25% High). A not significant diversity in the percentage of calves was observed for low (R^2^ = 0.08) to average (R^2^ = 0.24) BPI sires. However, the increase in R^2^ (0.86) showed there was a greater tendency (*P *= 0.02) for high BPI sires to have a greater percentage of grand calves born in the first 21 days as the percentage of their daughters born in the first cycle increased.

When looking at high performing bulls, it is important to consider how many calves are born in the first 21 days, as it is beneficial for both calf performance and dam reproductive efficiency. A study by Funston et al. (2012) reported that the calving cycle of when either heifer or steer calves are born, significantly impacts performance. The authors found that steer calves born in the first 21 days had increased weaning and hot carcass weight and improved carcass quality traits. For heifer calves born in the first 21 days, weaning and pre breeding weights were highest, and the percentage of heifers cycling at the beginning of the breeding season was 70% compared to 58% and 39% for second and third cycle born heifer calves, respectively (Funston et al., 2012). These are all economically important traits, and therefore bulls that produce more calves in the first cycle will have increased economic benefit to the herd and operation (Damiran et al., 2018, Drake et al., 2012).

### Performance of replacement heifers based on sires’ BPI groups

In the current study, 159 and 156 heifer calves were born from 16 and 14 sires in 2015 and 2016, respectively. As shown in Table 2, those sires were ranked into three bull groups based on their BPI values; however, replacement heifer selection by the owner was not based on a sire’s calculated BPI. Among heifers born, 74 animals from the year 2015 (on average ∼47% retention within each BPI group and ∼53% sold) and 86 animals from the year 2016 (on average ∼55% retention within each BPI group and ∼45% sold) were retained as replacements to produce and wean 160 and 171 calves, respectively, over four calf crops (2017 to 2020). In their first calving, 73% of the 2015 retained heifers weaned a calf, while the value was a bit higher (79%) for 2016 retained heifers. By the calving year 2020, only 29 replacement heifers (39.2%) born in 2015 remained in the cowherd. The 2016 born heifers had higher retention; 59.3% remained in the herd after weaning their third calf in 2020 (Table 2). Looking at each sire BPI group separately, the bottom 25% group showed a larger proportion of 2015 and 2016 born heifers remaining after several calving seasons than the other two groups. This might refer to the fact that as the number of females per sire increased in the Average and High BPI groups, the probability of open cows and culling rate would also increase. In other words, as low BPI sires are not under high pressure of production as Average and High BPI sires, they may produce more qualified females with larger probability of retention. Further investigation is required with larger data size. Considering similarity in the initial number of retained heifers born in 2015 and 2016 in the Bottom and Average BPI groups, and despite a larger increase in the number of initial retained 2016 heifers in the High BPI group (probably affected by the management decisions), 2016 born females overall showed higher retention than 2015 after several calvings. This might be a result of crossbreeding and hybrid vigor effects on increased fertility, calf survivability and cow longevity (Kirkpatrick, 2017) that will be discussed further in the next section.

**Table 2.** Performance of replacement heifers born in 2015-2016 from targeted sires (based on BPI) after four years of calving.

				Calves born to retained daughters								
Sire BPI Group (Avg BPI)	No. sires	No. daughters sired	No. daughters retained	2017	2018	2019	2020	% heifers remaining after several calvings^1^	Total calves weaned after several calvings	Avg No. of grand calves/sire after several calvings	Avg kg WW/calf	Total kg weaned/sire
**2015**												
**Bottom 25% (0.38)**	4	21	10	9	6	6	7	70	27	6.8	196.63	1337.08
**Average 50% (0.96)**	8	81	38	26	22	20	13	34.2	81	10.1	194.78	1967.28
**Top 25% (1.72)**	4	57	26	19	14	10	9	34.6	52	13	192.48	2502.24
**Total**	16	159	74	54	42	36	29	39.2	160	–	–	–
**2016**												
**Bottom 25% (0.34)**	4	12	7		7	6	6	86	16	4	205.94	823.76
**Average 50% (1.16)**	6	63	33		28	24	22	67	74	12.3	207.92	2557.42
**Top 25% (1.76)**	4	81	46		33	26	23	50	81	20.3	196.91	3997.27
**Total**	14	156	86		68	56	51	59.3	171	–	–	–

1 4 years of calf production were analyzed for 2015-born heifers and 3 calvings for the 2016-born.

Typically, the variation in grand-calf numbers per sire was influenced by a sire’s prolificacy and their daughters’ longevity. As the BPI score for a specific sire increased, the number of daughters sired, retained and produced grand calves also increased. Therefore, as opposed to 2015, there was a greater contribution of grand calves per sire based on the Average 50% and top 25% BPI values, produced from 2016 born heifers (Table 2). Accordingly, the total kg weaning weight (WW) of grand calves per sire in the top 25% BPI group was approximately 2-fold and 5-fold greater than the bottom 25% in 2015 and 2016, respectively and the latter had significantly (*P *< 0.01) the highest value in production. However, sire BPI groups didn’t have a meaningful impact on average kg WW per calf produced from their respective daughters. In fact, the ability of replacement heifers to raise a calf to the weaning point for multiple years was similar among the 3 sire prolificacy groups. The larger differences observed in kg of weaned calf per sire from 2016 born heifers might be related to crossbreeding effects that will be discussed in the next section. Overall, this variability in sire performance agrees with the findings in previous studies (Holroyd et al., 2002; Van Eenennaam et al., 2007; Domolewski, 2017; Domolewski, 2023) and can directly impact herd genetics and potential profitability in the long term, as replacement heifers remain in the herd and continue to produce calves.

### EnVigour HX™ results

During the 2018 calving season, 359 calves were tested with EnVigour HX™ to estimate their genomic breed composition and vigor scores. As indicated in Table 3, the number of tested progeny increased as their dams’ birth year increased from 2004 to 2016. Except for progeny from 2015 born dams, all had a similar range of genomic breed compositions with an average vigor score of 73% (SD = 8%). Also, the lowest vigor score (19%) was a calf from a 2015 born dam, while the highest score (86%) was a calf from a 2016 born dam.

**Table 3.** Descriptive statistics of average vigor score (VS) for progeny born in 2018 by dam birth year.

Dam Birth Year	No. of progeny born 2018	Avg VS for progeny born 2018	SD	min VS	max VS		
**2016**	72	0.72	0.12	0.29	0.86		
**2015**	49	0.45	0.16	0.19	0.76		
**2014**	26	0.72	0.12	0.29	0.82		
**2013**	45	0.73	0.09	0.41	0.81		
**2012**	39	0.75	0.08	0.42	0.82		
**2011**	38	0.71	0.13	0.37	0.82		
**2010**	30	0.73	0.1	0.42	0.82		
**2009**	12	0.77	0.05	0.62	0.82		
**2008**	21	0.73	0.11	0.39	0.83		
**2007**	12	0.78	0.02	0.75	0.82		
**2006**	10	0.79	0.03	0.75	0.84		
**2005**	2	0.78	0.04	0.75	0.81		
**2004**	3	0.81	0.02	0.79	0.83		
	359 (total)	0.73 (Avg)					
**Heifers born**	Avg VS (SD)	# total	# with VS	% with VS	% heifers 0.7-0.84 VS	% heifers 0.5-0.7 VS	% heifers <0.5 VS
**2015**	0.53 (0.17)	74	41	55.4	10	56	34
**2016**	0.65 (0.15)	88	57	64.8	51	24.5	24.5
**2018**	0.72 (0.12)	79	77	97.5	81	10	9
	sum	241	175	–	–	–	–

In this study, there were 4 Angus and 16 Beef Booster sires, primarily used in crosses with all the dams and the 2018 born heifers, with an average vigor score of 34% (SD = 9%) and 86% (SD = 2%), respectively. As shown in Table 3, among the retained replacement heifers in this study, 2015 born heifers had the lowest vigor score, on average 53% (SD = 17%), with only 10% of vigor scores above 70%. The reason was because these heifers were predominantly sired by Angus bulls and therefore had a higher than 50% Angus proportion in their breed composition (Table 4). Also, the lowest vigor score of their 2018 born calves (45%) was a result of backcrossing with Angus bulls. Accordingly, the integration of pure bulls might be related to the lower percentage of calf-crop born in the first 21 days of calving in 2015, 2018 and 2020 (Figure. 2). Between 2016 to 2018 born heifers, there was an increasing trend for vigor scores of animals, so that from 77 selected heifers born in 2018, 81% had vigor scores between 70% and 84% with an average score of 72% (SD = 12%) (Table 3). This implied the breeder’s interest in selection of crossbred females with the highest potential vigor scores. Moreover, the increased vigor score of heifers and their progeny was related to the integration of Beef Booster bulls in breeding seasons as the chief sires of daughters or heifers’ progeny.

**Table 4.** Average genomic breed proportions of heifers born in 2015-16 and 2018 in groups of 0.1 vigor score (VS) change (SD in parentheses) and their comparison with Basarab et al. study (2018), along with Angus and Beef Booster sires’ breed proportions and corresponding 2018 born progeny.

	VS	Angus	Gelbvieh	Hereford	Limousin	Charolais	Maine Anjou	Simmental	Holstein	Jersey	Shorthorn
**Heifers born 2015-16**	0.10-0.20	0.91 (0.01)	–	0.02 (0.03)	–	0.03 (0.07)	–	–	–	–	–
	0.20-0.30	0.85 (0.02)	0.01 (0.04)	0.05 (0.04)	–	–	–	–	–	–	–
	0.30-0.40	0.78 (0.02)	0.01 (0.03)	0.04 (0.06)	–	0.01 (0.04)	–	–	–	–	0.02 (0.05)
	0.40-0.50	0.70 (0.03)	–	0.10 (0.07)	0.01 (0.04)	0.01 (0.05)	0.01 (0.04)	–	–	–	0.02 (0.04)
	0.50-0.60	0.60 (0.08)	–	0.15 (0.09)	–	–	0.02 (0.05)	0.04 (0.07)	–	–	0.05 (0.06)
	0.60-0.70	0.48 (0.06)	0.04 (0.07)	0.20 (0.05)	0.02 (0.04)	0.02 (0.06)	0.01 (0.03)	0.01 (0.04)	–	–	0.01 (0.04)
	0.70-0.80	0.34 (0.06)	0.07 (0.09)	0.25 (0.05)	0.02 (0.04)	0.05 (0.08)	0.04 (0.07)	0.04 (0.08)	0.02 (0.04)	0.02 (0.02)	0.01 (0.03)
**Heifers born 2018**	0.30-0.60	0.74 (0.08)	–	0.06 (0.08)	–	–	–	0.02 (0.04)	–	–	–
	0.60-0.70	0.52 (0.02)	0.10 (0.02)	0.16 (0.02)	0.10 (0.02)	–	–	–	–	–	–
	0.70-0.80	0.41 (0.04)	0.09 (0.04)	0.20 (0.05)	0.04 (0.05)	0.02 (0.03)	–	0.04 (0.04)	–	–	–
	0.80-0.84	0.33 (0.04)	0.06 (0.04)	0.21 (0.05)	0.07 (0.04)	0.01 (0.03)	0.02 (0.04)	0.06 (0.06)	–	–	–
**No. Heifers with GBC^1^**	Avg (SD)										
**Current study (175)**	0.65 (0.16)	0.54 (0.19)	0.05 (0.06)	0.16 (0.09)	0.02 (0.04)	0.02 (0.04)	0.01 (0.03)	0.02 (0.05)	–	0.005 (0.01)	0.01 (0.04)
**Basarab et al. 2018 study (412)**	0.40 (0.17)	0.62 (0.28)	–	0.23 (0.23)	–	0.11 (0.21)	–	0.02 (0.03)	–	–	–
**Sires**											
**Beef booster**	0.83-0.88	0.17 (0.05)	0.13 (0.08)	0.23 (0.03)	0.06 (0.05)	0.03 (0.04)	0.01 (0.03)	0.09 (0.04)	0.004 (0.02)	0.04 (0.06)	0.02 (0.04)
**Angus**	0.21-0.41	0.80 (0.06)	–	0.02 (0.04)	–	–	–	0.02 (0.04)	–	–	–
**2018 born calves from 2015-16 heifers**	0.10-0.20	0.90 (0.007)	–	–	–	–	–	–	–	–	–
	0.20-0.30	0.85 (0.02)	–	–	–	–	–	–	–	–	–
	0.30-0.40	0.79 (0.02)	–	0.03 (0.04)	–	–	–	–	–	–	0.01 (0.03)
	0.40-0.50	0.72 (0.04)	–	0.10 (0.02)	–	–	–	0.01 (0.03)	–	–	–
	0.50-0.60	0.60 (0.03)	–	0.10 (0.04)	–	0.01 (0.03)	0.01 (0.02)	–	–	0.02 (0.04)	–
	0.60-0.70	0.50 (0.05)	0.006 (0.02)	0.17 (0.03)	0.003 (0.01)	0.007 (0.02)	0.003 (0.01)	0.004 (0.02)	0.003 (0.01)	0.05 (0.04)	0.02 (0.03)
	0.70-0.80	0.36 (0.05)	0.02 (0.03)	0.21 (0.04)	0.009 (0.02)	0.02 (0.04)	0.02 (0.03)	0.02 (0.03)	0.002 (0.01)	0.07 (0.03)	0.03 (0.04)
	>0.80	0.22 (0.02)	0.11 (0.006)	0.25 (0.04)	–	–	–	0.05 (0.06)	0.03 (0.03)	0.07 (0.01)	0.08 (0.05)

1 Genomic Breed Composition.

One minus the summation of breed proportions in each row is equal to the indeterminate proportion (not shown).

The high vigor score females (>48.6%) in Basarab et al. (2018) study had a 46.8% retention to third parity with a reduction to 33.5% in fourth parturition. Similar results were observed for 2015 born heifers in the current study, showing an average vigor score of 53% with a reduction in retention to 48.6% and 39.2% in third and fourth parities (*P *< 0.01), respectively (Table 2). The reason was because Basarab et al. (2018) used cow herd data in which crossbred heifers were mainly mated to purebred bulls. A similar case occurred for the 2015 born heifers bred to Angus bulls in the current study. However, the longevity to third parity in 2016 born heifers was overall increased to 59.3%. This might be because of the utilization of higher vigor score females (on average 65%) and using crossbred Beef Booster bulls as their sires (*P *< 0.05). Although there were only two parities of data for 2018 born heifers (not shown), they also revealed a greater increase in longevity to second parity compared to 2015-16 born heifers (87% vs. 57% and 65%) due to the utilization of female replacements with a higher average vigor score of 72% and Beef Booster bulls as their sires (*P *< 0.05). Retention of replacement females is of major importance for sustainability and profitability on beef operations (Cushman et al., 2013). Each year, a sire contributes 50% of the genetic make-up of the calf crop, and if a producer retains their own replacement heifers, a prolific sire’s influence will become even higher if they also have a large vigor score (Dhuyvetter et al., 1996). This means Beef Booster (composite breed) sires that can produce high quality replacement heifers in the herd would be more economically beneficial relative to purebred Angus bulls.

Table 4 categorized the vigor score results of heifers, sires and progeny into groups of 10% change. It showed their genomic breed composition diversity in terms of the number and predicted fraction of founder breeds when the vigor score increased from 10% to more than 80 percent. Among the sires used by the cooperating herd, Beef Booster bulls typically had much larger vigor scores than purebred Angus bulls, so that their genomic composition only included 17% Angus as a founder on average. Moreover, based on their founder breed proportion averages, they indicated to be primarily a mixture of M2, M1 and M4 along with some percentage of M3 and TX strains, more likely suitable for breeding programs on cow-calf operations. In terms of heifers born in 2015 to 2018 (Tables 3 and 4), as the percentage of animals toward a vigor score higher than 50% increased, there was a continuous decrease in Angus proportion (the predominant breed) but an increase in Hereford contribution due to the incorporation of Beef Booster in mating seasons. Moreover, a larger introgression of Gelbvieh, Limousin and Simmental occurred in 2018 born heifers, while there was a reduction in Charolais, Maine Anjou and dairy breeds, which might reflect the breeder’s objective in producing heifers with more growth capacity by selection of specific sire lines. Also, looking at 2018 born calves from 2015-16 born heifers demonstrated the effect of M3 sire strains by increasing Jersey and Shorthorn contributions to improve milk production and easy calving features. Overall, compared to Basarab et al. (2018) results (Table 4), heifers in this study showed a substantial increase in their average vigor score (0.65 vs. 0.40) due to a more intense selection of higher vigor score females. In addition, as opposed to the selection of purebred bulls in the Basarab et al. (2018) study, using crossbred sires in this study resulted in a reduction of Angus, Hereford and Charolais contribution with some introgression of Gelbvieh, Limousin, Maine Anjou, Jersey and Shorthorn (Table 4).

**Fig. 5.**
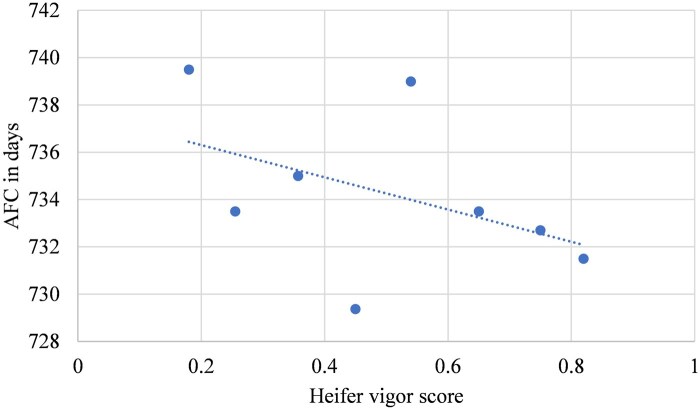
The relationship between AFC and vigor score of crossbred heifers born from 2015 to 2018.

**Fig. 6.**
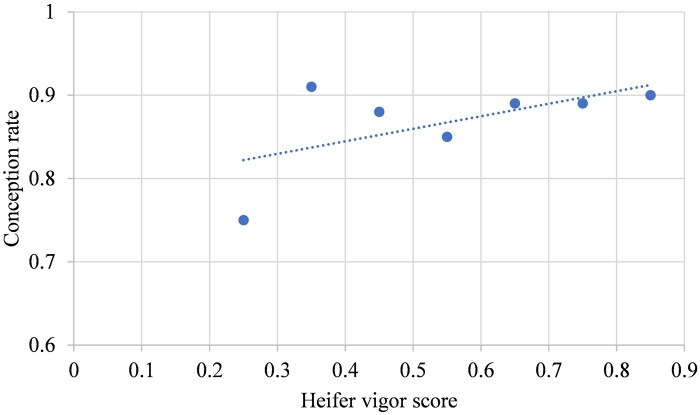
The relationship between conception rate and vigor score of crossbred heifers born from 2015 to 2018.

**Fig. 7.**
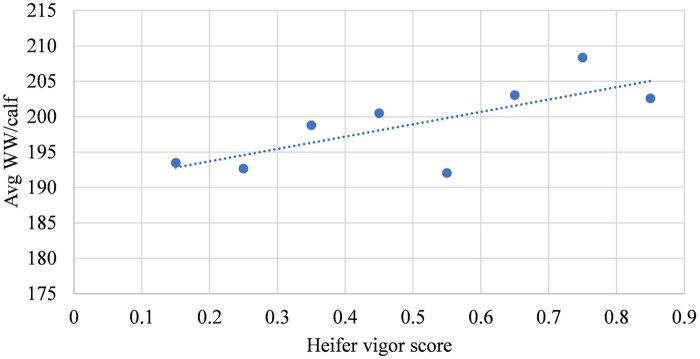
The relationship between the average kg WW per calf and vigor score of crossbred heifers born in 2015-2016 after three parities.

**Fig. 8.**
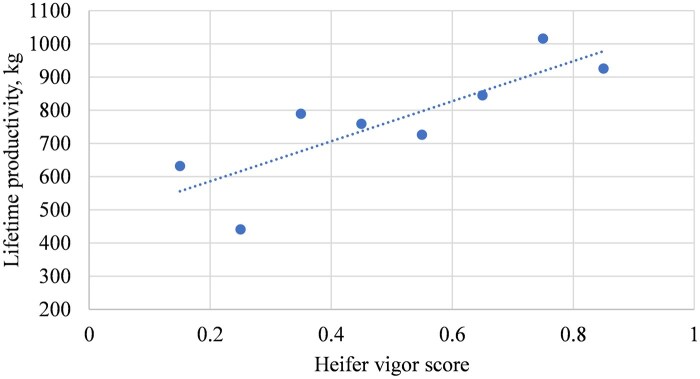
The relationship between heifers’ lifetime productivity and their vigor scores over four parities.

The linear effects of heifers’ vigor score based on groups of 10% change on their AFC, conception rate, average WW per calf and total lifetime productivity (cumulative calf WW per cow after several parities) are depicted in Figures 5 to 8, respectively. The results of the current study indicated that heifers were on average at an older age of days at first calving relative to Basarab et al. (2018). Furthermore, only a 0.7-day reduction was observed in AFC per 10% increase in heifers’ vigor score (Figure. 5), which was not statistically significant (*P *= 0.26). This might be due to the addition of continental breeds like Gelbvieh, Limousin and Simmental in the females’ genomic breed composition that resulted in heifers attaining puberty later than British breeds such as Hereford and Angus (Stuttgen, 2021). On average, the heifers in the current study achieved a greater conception rate compared to Basarab et al. (2018). The improved conception rate could be a result of higher vigor scores among retained replacement females (Figure. 6). However, despite a similar ∼2% increase in conception rate, it was not meaningful (*P *= 0.16) per 10% increase in vigor score. This might be related to the smaller sample size of this study with lower variation of vigor scores (Table 3 and 4). Although the average WW per calf was less than reported by Basarab et al. (2018) (Figure. 7), it showed a slight increase of 1.74 kg per 10% increase in heifers’ vigor scores with a smaller *P* value (0.13). Finally, greater cow lifetime productivity was observed in the current study over four parities (Figure. 8) with a 60.3 kg increase per 10% increase in heifers’ vigor scores. This might suggest the importance of economic benefits from heterosis when using both crossbred bulls and females.

Gregory et al. (1982) indicated that targeting contributions of more than four to eight breeds in the composition of crossbred animals resulted in a range of 75 to 88% vigor scores. However, the efficiency of this increase in vigor scores by heterosis would only be less than 1% increase on the production trait of weight weaned per cow exposed. The results of this study demonstrated the interest of breeders in selection of heifers with higher than 75% vigor score. However, excluding such heifers from the respective Figures. 5 to 8 did not make a significant change in the linear regression results (not shown). In other words, the difference in fertility and production capacity was not meaningful when adding such females in the mating plans. This implies selection of heifers with a vigor score higher than 75% (as in the current study) will not warrant a significant change in the breeder’s enterprise productivity. Moreover, it is important to mention that adding founder breeds of less than 10% genomic contribution to the crossbred composition of animals to increase their vigor score above 75% will not necessarily benefit from targeted traits in those founder breeds. In fact, retaining such females will not change the overall productivity unless there is a specific genetic selection program for those traits of interest from each specific founder breed (Khorshidi, 2022). Therefore, a 75% cutoff is suggested as the highest economically sufficient vigor score when using EnVigour HX™ test results to select for crossbred females.

## Conclusions

Optimizing breeding strategies and reproductive performance is essential for genetic improvement in the commercial beef herd. Nowadays, DNA parentage testing is gradually being adopted by commercial cow calf producers in multi-sire breeding pastures to evaluate the prolificacy of sires or aid in culling decisions of non-prolific bulls. This is because the prolificacy of a sire essentially determines their long-term contribution to the profitability and genetic composition of the herd, and this is accentuated when their heifer calves are also retained as replacements within the herd. In conclusion, the BPI results of this study approved the importance of DNA parentage testing in multi-sire breeding pastures. It not only identifies high prolific sires but also helps decide early culling for low fertile bulls due to moderate repeatability of sire’s prolificacy across years. This also has a direct impact on the overall weaned production and the number of calves born in the first 21 days of calving period.

The benefits from crossbreeding are clear and have been well established for many years in the beef cattle industry. The use of vigor score testing may help support management decisions on improving specific traits based on certain genetics available in the herd. In conclusion, the repeatability of prolificacy of Beef Booster sires may increase due to the effects of heterosis. In addition, mating sires with higher vigor scores to crossbred heifers may result in lower age at first calving or higher production efficiency than EnVigour HX™ suggestions. This means genomic breed composition of crossbred animals based on founder breeds should be considered in mating plans.

Optimizing heterosis can result in female progeny with improved reproduction and lifetime productivity. However, it would be important to consider how long results from these genomic tests are used before their use may not provide any more significant improvement in breeding outcomes. Therefore, it is recommended to consider up to an average vigor score of 75% to economically retain the efficiency of crossbred females in fertility and production traits.

## Notes

1. In 2020, Neogen acquired Delta Genomics and incorporated the EnVigor test into its Igenity^®^ suite of tests (Drovers, 2020).

## List of Abbreviations

AFC: age at first calving
BPI: bull prolificacy index
SNP: single nucleotide polymorphism
WW: weaning weight
